# Green and sustainable City will become the development objective of China’s Low Carbon City in future

**DOI:** 10.1186/2052-336X-12-34

**Published:** 2014-01-14

**Authors:** Liu Li-qun, Liu Chun-xia, Gao Yun-guang

**Affiliations:** 1 College of electronic and Information engineering, Taiyuan University of Science & Technology, Taiyuan, China; 2 College of computer science and technology, Taiyuan University of Science & Technology, Taiyuan, China

**Keywords:** Energy saving, Low carbon city, Sustainable development

## Abstract

Environmental pollution and greenhouse gas emissions are becoming significant environmental issues in China, thus the sustainable development and revival of the country is impossible using the conventional path of encouraging economic growth at the expense of the environment. In response to the global warming, the prices of the traditional energy rise considerably, and a series of environmental problems, China must improve its own mode of economic development. Hundreds of Chinese cities have billions of square meters of buildings and most industry and the annual energy demand is an astronomical figure. China’s government is facing increasing pressure in the low carbon international backdrop, and the low carbon city becomes the inevitable developmental direction of Chinese city in the foreseeable future. The description is first centered on energy structure/energy consumption per unit/urbanized status, and urban energy consumption status, and then concerned with the efforts and measures of Chinese government, to realize the energy saving. Finally, we present the developmental prospect and barriers and the promotion measures related to the low carbon city under the government policy, financial incentives and funding supports, etc.

## Introduction

It is well known that China is the largest developing country in the world. Since 1978, Chinese government has begun its open-door policy and economic reforms, China has experienced spectacular economic growth, and hundreds of millions of the ordinary people have been raised out of poverty [1]. China has become the world’s second largest economy in 2010. With the nation’s rapid industrialization and social development, the annual energy demand ranks the second in the whole world, just behind the USA. The total energy consumption in 2010 was more than 3 billion tons of standard coal. Environmental pollution and greenhouse gas emissions are becoming significant environmental issues in the country.

**Figure 1:**
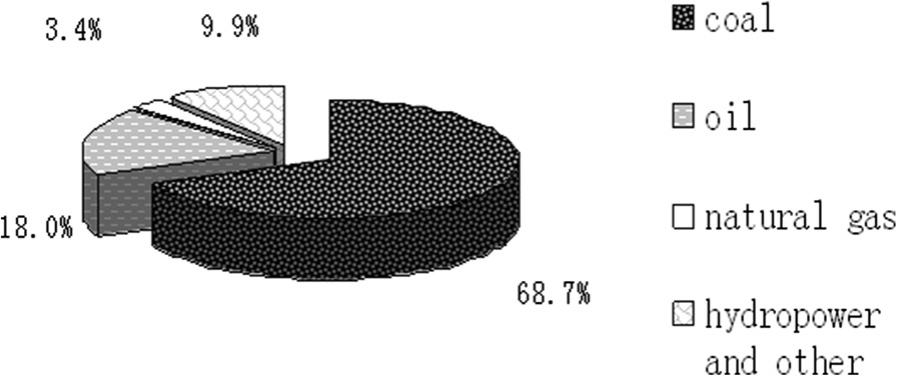
**Energy structure of China in 2009 (Source: [**[2][10][11]**]).**

**Figure 2:**
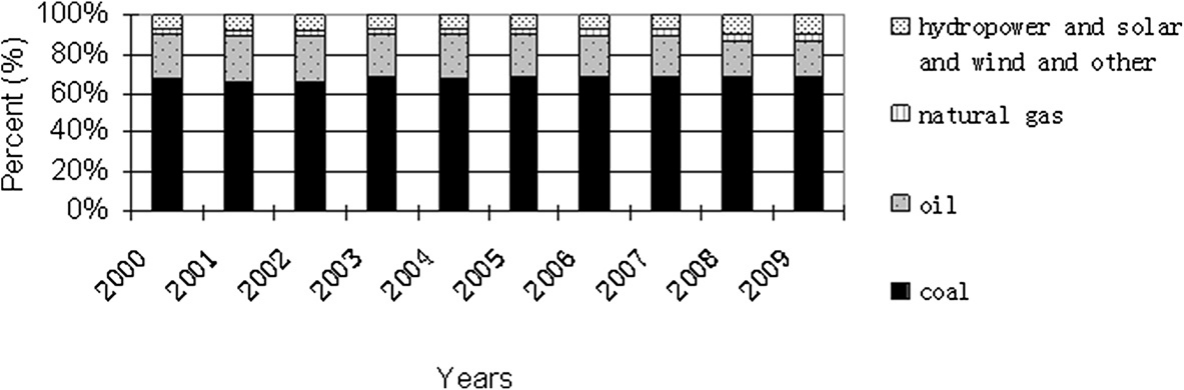
**China’s energy structure in the past decade (Source:[**[2][3]**]).**

**Figure 3:**
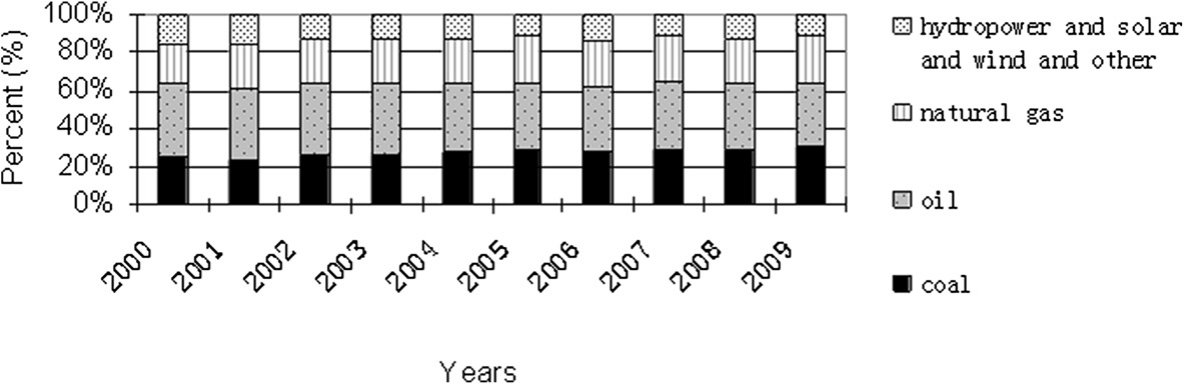
**Global energy structure in the past ten years (Source: [**[4]**]).**

**Figure 4:**
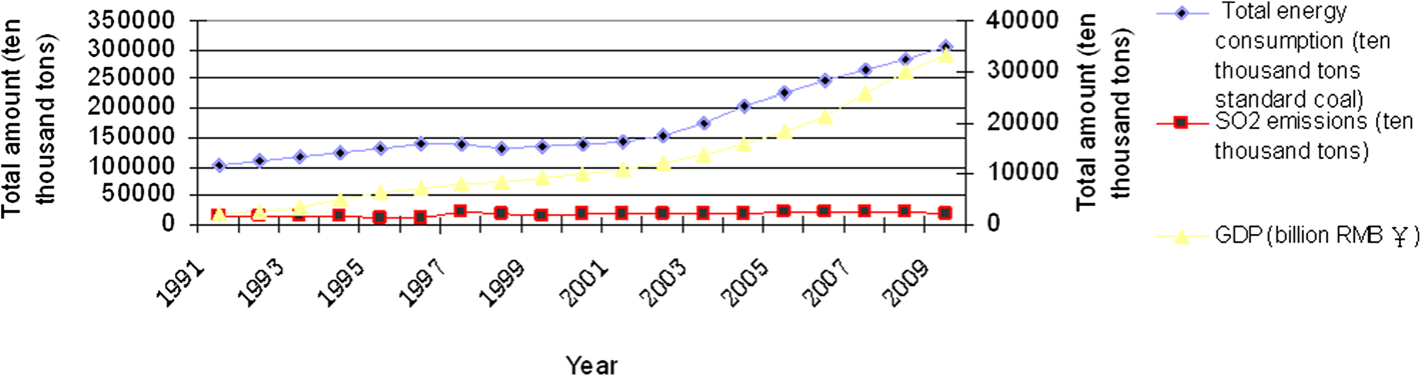
**China’s total amount of energy consumption and SO2 and CO2 emissions in the past twenty years (Source: [**[12][14]**]).**

For example, China is heavily depending on dirty-burning fossil resources to fuel its rapidly growing economy. The share of fossil fuel resources in the whole energy structure accounts for about 90% in the past three decades. (Figure 1) shows the energy structure of China in 2009, and the shares of coal, oil and natural gas are 68.7%, 18% and 3.4%, respectively, and the renewable energy and nuclear energy has a share of 9.9%. As shown in Figure 2, the dirty-burning coal to fuel in whole energy structure does not have any changing in the past decade, and the main changing is that the share of hydropower and renewable and nuclear energy increases from 6.7% to 9.9%. As a conclusion, China’s energy structure very inappropriate as compared with the world’s energy structure over the same period. The shares of oil, natural gas, coal and others in 2009 were about 34%, 24%, 30% and 12%, respectively [2,3], which can be seen from Figure 3[4]. The dirty-burning coal to fuel has brought a series of environment questions such as acid rain, water pollution and soil pollution. For example, the total consumption amount of coal and oil were more than 2.74 and 0.36 billion tons in 2008, respectively, and natural gas was about 80.7 billion *m*^*3*^[5]. The tremendous energy consumption induces the high strength carbon emissions, i.e., the SO2 emission from 2000 in China is more than 20 million tons, which ranks the first in the world [6], and the CO2 emission is more than 4.5 billion tons, which ranks the second in the world [7], and the total pollution loss accounts for 10% of Chinese gross domestic product (GDP) [8]. The total amount of energy consumption and SO2 and CO2 emissions in the past twenty years can be seen from Figure 4. China achieved more than 15 times the Gross Domestic Product (GDP) with only treble energy consumption during the period 1991–2009, and the total emissions of SO2 have decreased since 2005. Figure 4 depicts that the maximum value of SO2 emissions appeared in 2005 and is about 25.49 million tons. The total area of acid rain is more than one of three of the national area. China’s CO2 emissions roughly experienced three stages during the period 1990–2006: the first stage: 1990–1996. This one phase carbon dioxide emissions by low growth, from 2.415 to 3.395 billion tons, the average growth rate is about 6.77%. The second stage: 1997–2002. This one phase rate of CO2 emissions by 33.55 million tons, relatively flat rose to 37.02 million tons, the average growth rate for 2.07%. The third stage: 2003–2006. This one phase carbon dioxide growth faster, by 43.53 million tons to rise to the 56.26 million tons, the average growth of 13.4%. But in 2006 has eased growth rate fell to 8.5 percent [9].

**Figure 5:**
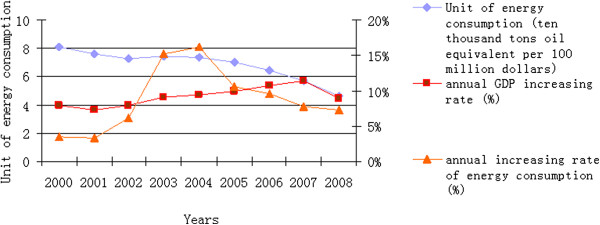
**China’s unit of energy consumption of GDP, annual GDP growth rate and annual energy consumption growth rate in the past nine years (Source: [**[17]**]).**

The operation of modern industrial society needs enough electric power supply, and the electric power for every country has become a powerful engine. As the second economic entity in the world, the cumulative installed capacity of electric power system in China is about 0.962 billion kilowatts, and the annual growth is about 13.22% in past five years, and the annual installed capacity of electric power system is about 0.1 billion kilowatts in recent years. Here, the installed capacity of fossil resources has a share of 75%, and the power efficiency is less than that in the developed country [15]. The total consumption of Chinese top ten power groups accounted for one fifth of national coal output, and the dirty-burning coal to fuel caused by environmental loss as high as 87 billion Renminbi (REM, ￥). The climate damage of China’s power industry is more serious than that in the developed countries. For example, the emission of CO2 per kilowatt-hour in Japanese industry is about 418 g/kWh. While the emission of top ten power industries in China is more than 773.8 g/kWh, which is about 1.8 times of that in Japan [16]. In Figure 5, the changes of energy consumption per unit of GDP and the GDP growth rate in the past nine years are illustrated. The energy consumption per unit has decreased from 8.11 tons to 4.63 tons per ten thousand dollars during the period 2000–2008. At the same time, the annual growth rate of energy consumption is more than that of the GDP from 2003 to 2005. The total energy consumption in 2009 is about 2.2 times as compare with that in 2000, up 1.66 billion tons of standard coal form 2000.

In response to the balance between economic development, great energy consumption and environment protection, the traditional developmental model must be changed in order to achieve the sustainable development in the future. China’s urban energy status will be introduced in the next chapter. And then concerned with own efforts towards energy saving and pollutants cutting of China’s unilateral actions is discussed. Finally, the developmental prospect and barriers and recommendations of low carbon city are introduced, respectively.

### China’s urban development and energy consumption status

The rural economy is turning into the urbanized economy due to the nationwide urbanization, which is the inevitable result of national industrialization. On the one hand, this accelerates the mechanization of agricultural production and improves the agricultural productivity and provides a large number of employment opportunities for rural surplus labour since the industrial expansion. On the other hand, the backward development of the countryside is disadvantageous to the development of urban areas, which will affect the entire national economy development. The rural industrialization production is positive for the rural regional economy and the entire national economy development [18].

Chinese government has begun its open-door policy and economic reforms since 1978, and China has gradually unloosened the strict control of floating population. At present, there are billions of migrant workers have came into the city, and the urbanized process is speeded. The total amount of the migrant workers is more than 0.2 billion. The national urban population proportion has a share of 46%, namely, there are more than 0.6 billion people live in the city, however, which are less than that in industrialized countries with 20%.

Today, there are hundreds of large and medium-sized cities in China, and the population per city is more than 100 thousands. There are quite a number of rural population migration will come into the large and medium-sized cities in foreseeable future, which will become the stamina of the sustainable economic development. The fundamental reasons are that the migrant people needs working for the survival of himself and family, and the families need a lot of accommodations, and children need to get education, etc. Therefore, a common phenomenon in China is that there are more and more migrant workers worked and studied and lived in cities, and finally become a city people. For example, a huge number of migrant workers are working in the building industry, service business, mining enterprises, and various industries. With ever-increasing population migration from countryside to cities, the level of urbanization will continue enhancement and the city scale will constant expand and the urban population will unceasingly increase. If in the next 20 years, China’s urbanization reaches the share of 65%, which means that there are more than 0.25 billion rural populations will come into cities, and the urbanized process holds the enormous economic benefits.

The city’s GDP in China accounts for about 85% of the whole GDP, and the annual energy consumption of are huge because of there are the numerous urban populations, enterprises and government units, namely, sufficient energy and resources are the engine for city operation. However, China’s cities, most of them are facing the energy and resources shortage, such as there are more than 400 cities is facing in dehydration, especially, in north district, the water resource is more serious than that in south district. With the rapid economic development, sufficient power supply in many Chinese cities cannot be met. For example, Shanxi Province is a very important coal production province, and the annual coal production amount is more than 4 million tons, but it have to face the electricity shortages in recent years also, and the gap of electricity is more than 25%. At the same time, with the accelerating urbanization, the urban population and scale is ever increasing, the safety management and emergency response have became a thorniest problem. For example, China’s urban drainage system was built many years ago, which is old and bad efficiency. The annual rainy season, once the city faces the rainstorms, and the mostly cities will become the swamp, such as Beijing and Guangzhou. At the same time, the number of the high-rise buildings in Chinese city incredibly increases in past two decades, and the safety emergency measures of the high-rise building cannot be ignored, such as a high-rise buildings fire happened in 2010, which took 58 people’s life in Shanghai.

Chinese GDP in 2010 has more than 5 thousand billions and just behind USA, and ranks in the second in the world. But an unavoidable issue is that the heavy industry development is very fast and the annual energy consumption is so high that the strong growth of energy consumption [19]. The annual new installed capacity of power generating is about 0.1 billion kW in the past five years. But the electricity shortage in the national scope is an inevitable question. Especially, the limiting power supply is very common in summer peak period. According to the statistical data in 2009, the GDP of second and third industry account for 46.3% and 43.4%, respectively. The share of the third industry is lower than that of the world’s average level with more than 20%, and the share of the second industry is far higher than that of the average level in the world. The share of the total industrial output value of heavy industry increases from 60.2% to 70.5% during the period 2000–2009, which is more than that in Japan, Germany, United States, and other. The total industrial energy consumption accounted for 71.3% of the total national energy consumption, while high energy-consuming industry accounted for about 80% of the total industrial energy consumption of heavy industry, namely, which has a share of more than 56%. At the same time, the industrial capacity is superabundant in nationwide, such as China’s annual steel output has reached nearly 0.6 billion tons [20].

Besides, the total energy consumption of China’s building accounts for about 20.7% of the total social terminal energy consumption. The total energy consumption of the northern urban building heating and rural life in 2004 is about 0.16 billion tons of standard coal. If architectural electricity and other types building energy consumption (e.g. cooking, lighting, home appliances, living hot water, etc.) are converted into electricity, which are approximately 550 billion kWh per year and accounts for 27% ~ 29% of the total social terminal energy consumption [21]. According to expert’s calculated data, the average standby energy consumption of urban family has accounted for about 10% of the total energy consumption, which equal to a single 30 watts ever-burning lamp. If the energy consumption of the building materials production process is considered, and the construction-related energy consumption accounts for 46% of the whole society, Now, China’s annual new-built building is about 2 billion square meters, and most of building is the high energy-intensive architecture which has a share of 80%. The existing building is about 43 billion square meters and only 4% adopted energy efficiency measures, and the heating energy consumption per unit in building area is more than three times of the developed countries new-built buildings. According to statistical data, if don’t take strong measures by 2020, the building energy consumption will become 3 times of that of current status. The steel consumption of building is about 55 kilograms per square meter, which is higher than that in the developed countries with 10% ~ 25%, and the cement consumption per square meter is about 221.5 kilograms, and the cement consumption of every cubic meters of concrete is more than 80 kilograms as compare with that in the developed countries. Based on the land occupation, urban per capita land in the developed country is about 82.4 square meters and in developing countries is average 83.3 square meters, however, the amount in China is more than 133 square meters. At the same time, the resource consumption of building during the process using the same technology is about 2 ~ 3 times to compare with the developed countries. The water-consumption is higher than that of the developed countries with more than 30%. Current trends by 2020, the building energy consumption in China will reach 1.09 billion tons of standard coal. According to Chinese generating cost, every ton of standard coal is approximately equal to 2700 kWh electric energy, that is, China’s building energy consumption in 2020 will reach 2943 billion kWh, which is more than the total generating capacity of Three Gorges hydropower station during 34 years [22].

China’s economy has more than thirty years of rapid development since 1978, and the common people’s living standard is ever-increasing and the ordinary people of transportation also have a huge change. Thirty years ago, the ordinary people of transportation are primarily dependent on bike and public transportation, but now there are various ways of travel, such as planes, private cars and public transportation modes, etc. But rapidly rising proportion of planes and private cars travel inevitably caused the rapid growth of energy consumption, large scale greenhouse gas emissions, large environmental pollution and traffic congestion. For some actual example, China’s traffic volume of plane passenger in 2010 has breakthrough 0.266 billion person-times, and the growth speed ranked in the first of the global airline industry [23]. The private car amount in 2008 has more than 40 millions. With the rapid increasing amount of private cars, there are more and more pressure in urban transportation, such as Beijing, Shanghai, and Guangzhou and so on. At the same time, the lifestyle of ordinary people has changed in all fields, not only in eating and drinking, playing, and clothing, but also in people’s minds. Today, some Chinese people have formed the high consumption and energy-guzzling life style.

In the order to achieve the low carbon economy and sustainable development in future, the existing industry development mode and lifestyle must be improved. In other words, China’s each aspect must change the existing ways of development like industrial, building, lifestyle, etc.

### China’s efforts and measures towards energy saving

In recent years, Chinese authorities continuously promoted the policy of energy conservation and emission reduction during the significant opportunity period of international financial crisis, to accelerate the economic structure adjustment and create a good environment for energy conservation and emissions reduction. The Chinese government in the 11th five-year plan explicitly proposed that GDP per unit reduces the energy consumption by 20% and the emissions reduction of selected pollutants with 10%. This means that an average annual GDP per unit will be reduced the energy consumption by 4%, just may successfully completed on schedule objectives. According to the statistical data from 2006 to 2008, China had discontinued some backward productions such as the small-scale power unit with 38.26 million kilowatts, the backward iron capacity with 60.59 million tons, the steel with 43.47 million tons, and the cement production capacity with 0.14 billion tons. The energy-saving management of the key energy-using enterprises is strengthened and the energy-saving action of 1000 big enterprises is managed and the energy-saving of architecture, traffic and public institutions is promoted. As mentioned above, to the implementation of energy saving and emission reduction, a series of policies and measures have be established by Chinese government, and some significant results have achieved. According to statistical data, the national GDP energy consumption per unit declines by 1.79% in 2006, by 4.04% in 2007, by 4.59% in 2008, and by around 5% in 2009 [24].

Chinese central government and local governments have regarded the great importance of the building energy efficiency improvement such as the study of building energy efficiency breakthrough, optimize limited resources, and promote Chinese building energy saving which has caused some significant progresses. Some specific measures include: I) The central heating metering reform, to promote the building insulation and to encourage the behaviours of energy-saving, the conventional heating measurement based on the area is changed. II) Scientific planning construction energy conservation work located in the southern region, the key is to improve the thermal insulation of building. III) The development and popularization of low energy consumption and large public construction technology, the construction area of large-scale public buildings has a share of 4% of the total urban building, which accounts for 20% of the total energy consumption of urban building. The low-energy technology for the large-scale public buildings should be explored, which can greatly alleviate the nervous conditions of urban electricity supply due to the rapid growth of the medium-large public buildings. IV) The technological progress of building materials, such as the hollow bricks is used to replace the original solid brick, the wide use of energy saving materials, and the energy consumption of building materials is reduced during the production process, etc. V) Increasing the amount of renewable building, such as the photovoltaic (PV) building, wind power building, and hybrid wind-solar building, etc. [21].

At the same time, Chinese government have carried out some efforts towards energy saving for the industrial and mining enterprises. Fore example, the national development and reform commission (NDARC) has noted nine key energy industries which includes the steel, nonferrous metals, coal, electricity, petroleum, petrochemical, chemical industry, building materials, textiles, and manufacture. There are 1,000 enterprises since 2006 are selected to carry on the energy saving of the detection and the energy consumption of every enterprise is more than 180 thousand tons of standard coal because of the energy consumption of these enterprises accounted for 60% of that in the total industrial users, that is, which has a share of 40%-50% in the terminal energy consumption [25]. NDARC has established some specific measures which include: 1) Effective control the over-rapid growth of high energy consumption and high pollution industries. These newly-built high energy consumption and high pollution projects are strictly control, such as the supplies of land and credit are reduced and the market admittance threshold are raised. 2) Accelerate the elimination of backward production capacity. 3) Full implementation of the energy conservation and emission reduction key projects such as the saving and replacement of petroleum, coal boiler renovation, cogeneration, building energy saving, SO2 control of coal-fired power plant, and the construction and renovation of urban sewage treatment plants. 4) Improving the energy saving and emission reduction of key enterprise. The governments at all levels should intensify the inspection and guidance of key enterprise, and implement strict rewards and punishments measures. 5) Accelerating saving the progress of science and technology. The government has organized some special actions for the implementation of energy saving and emission reduction technology. The technical reformations of enterprises are encouraged and supported to conserve energy and reduce emissions. 6) Developing the recycling economy such as the improvement of the mineral resources comprehensive utilization, the solid waste comprehensive utilization, renewable resources recycling, water resources recycling, and refuse resources utilization and free-pollution disposal, etc. 7) Perfect policy system. The price reformation of the natural gas, water and heat resource products should be timely promoted, and the charges standard of pollution emissions should be increased. 8) The investment of energy saving and emission reduction should be increased. The energy conservation and emission reduction mechanism consists of the government guidance, the participation of enterprise and society, to promote the development of the enterprise pollution regulation, ecological restoration, and environmental protection. 9) The legal construction of energy conservation and emissions reduction should be strengthening. 10) The supervision and administration of energy conservation and emission reduction should be strengthening. The perfect index system and monitoring system and assessment system of the energy conservation and emission reduction should be established, to ensure to gain the real data. Certainly, the strict enforcement regulation should be established. The constantly monitor of the key energy-using units and sources should be strengthening [25].

Furthermore, the transportation industry is the second-largest oil consumption industry and just behind manufacturing industry in China, which is the key industry of the energy conservation and emission reduction because of the oil consumption of the transportation industry accounts for about 33% of that of whole society. The Chinese government encourage measures include: the statistical and monitoring and assessing systems of industry of energy saving and emission reduction are established, and the admittance system of vehicles fuel consumption is formulated, and the new energy public transportation is promoted, and so on. Although the fuel consumption of automobile in China reduced by 10% or more in the nearly decade, but the utilization efficiency is obviously low, which is more 25%, 20%, 10% than that in Europe, Japanese, and United States, respectively. Specially, 100 ton-km fuel consumption of truck is twice times of foreign advanced level. Thus the energy-saving technology of transport vehicle should be improved. Some specific measures have been implemented. For example, I) The department of transportation has carried out the exited work of the high fuel consumption vehicles, to reached the limit standard of fuel consumption for full high fuel consumption vehicles in five years time and to ban the high fuel consumption vehicles into the road transport market in future. II) Popularization of new energy public transportation such as the project of 10 thousand vehicles in one hundred cities has been promoted by using hybrid energy-saving and new energy buses and taxis. III) The construction of the Changjiang river waterway as the key point of inland water transport is accelerated to promote the ship standardization and to give full play the comparative advantages such as the low energy consumption, low pollution, and occupies little space. IV) Increasing the investment in the public transportation, and some projects of the subway and rail transit in big cities had been established, and the number of bus route had been increased. V) The investment in traffic has increased to promote the energy conservation and emission reduction [26].

Besides, the ecological effect of urban landscape can absorb all kinds of pollutants and reduce the heat island effect and dust fall and minus noise, and avail to purify the air and conserve water, etc. Furthermore, the energy consumption of city has a share of 80% of that of nation, and the shares of pollution emission and greenhouse gas emissions are 95% and 80%, respectively. It is very important to conserve energy and reduce emission in the key cities due to the urban landscape is the key of the energy conservation and emissions reduction. Some laws has been established by Chinese government, such as the greening lands area of new city should has a share of 30% or more of the total land area and the share in old city shall not be lower than the total land area of 25%. Today, the share in some cities has more than 40% like Shenzhen, Nanjing, and Dalian and so on [27]. The greening lands area in city has reached 135.65 million hectares till 2009, and the green coverage has a share of 37.37%. By the end of 2009, the park greenbelts area in city has reached 9.71 square meters per capita [28].

The PV and wind power market in China has come into the rapid increasing stage over the last years, and the reduction of PV cell and wind power system price can response this trend. Today, Chinese government regards the development in renewable energy field, and billions of dollars has been invested in the wind power and PV and nuclear power field, and the total amount is more than 400 billion dollars. The annual growth rate of new installed capacity in past six years is more than 100% [29]. China has become the biggest manufacturing country of solar energy cell from 2007, and the most famous manufacturer in China is Wuxi Shangde. There are some large-scale grid-connected PV and wind power projects have been established in Western China. As mention above, China’s government have implemented some efforts and measures towards energy saving, and the Chinese government will adopt more effective measures in future in order to realize the sustainable development and low carbon society.

### Developmental prospect of Low Carbon City in future China

There are some environmental questions have been threatened to the human survival and development such as global warming, rising sea levels, living beings extinction, melting glaciers and extreme weather, etc. There are only one Earth, we are not only satisfy the survival and development of ourselves but also should consider the development of future generations. The development ways of high pollution and high energy consumption and high emissions since the industrial revolution must be rejected, and it is not only the responsibility of developed countries, but also the responsibility of developing countries. Low carbon city has become the world’s common pursuit, which pay attention to the ecological cost minimization and the harmony between human and nature in the economical developmental process.

Chinese attitude for the global climate change is positive, and the Chinese government is actively implementing the low carbon economical strategy, and the prospect of the low carbon economic in China is optimistic. For example, 2009 September, President Hu Jintao, at the UN climate change summit, put forward China’s future climate change concrete measures: one is to strengthen the saving energy and improve energy efficiency, and the CO2 emissions reduction of GDP per unit has a share of 40%-45% from 2005 to 2020. The other one is to develop the renewable energy sources and nuclear power, and the non fossil energy accounts for approximately 15% till 2020. Third is that the forest gather carbon is greatly increased, and the forest area till 2020 will more than increase 40 million hectares and the forest volume till 2020 will add 13 billion cubic meters more than that in 2005. The fourth is that the green economy is developed, and the climate friendly technology is actively developed to promote the low carbon economy and circular economy [30].

Today, many Chinese local governments have established the developmental goal of low carbon city. In order to realize the sustainable development of the city, the local governments have proposed many low carbon urban developmental goals, such as garden city, Chinese electric valley, and Sun City, etc. For example, BaoDing located in Hebei province, has formed photoelectric, wind power and power saving, store the electricity, transmission and power automation of the six major industrial system, and the renewable energy enterprises have more than 160.

The local government in BaoDing has established the developmental planning in future decade, and the total sales revenue will exceed 100 billion RMB and form the international renewable energy and power equipment industrial base, to explore the energy sustainable developmental road and make BaoDing truly become China’s low carbon advocates of economic development.

Furthermore, in order to achieve the developmental target of low carbon city, the Chinese government and local governments have increase the investment in transportation field such as the subway and the urban rail facilities which located in Beijing, Shanghai, Shenzhen and other first-tier cities. Some second-tier cities also is establishing the subway and the urban rail facilities to improve the public transportation ratios for common people and achieve the low carbon city traffic, like Xian, Changsha, Dalian, etc. Moreover, China has the world’s largest bicycle possession, and the Chinese government has tightened the propaganda of low-carbon lifestyle, there are more and more common people choose bikes as transportation at present.

Besides, China has the world’s largest artificial planting activities, and the total person-times of voluntary planting trees have more than 11.52 billion till 2008, who plants more than 53.85 billion trees. The area of planting trees in 2008 completed 4.771 million hectares and had an annual growth rate of 22.1%. Today, the green rates in many Chinese cities have more than 40%, like Shenzhen, Nanjing, and Dalian, and so on [27]. The greening covering area in city has reached 135.65 million hectares till 2009 and the green coverage accounted for about 37.37%. By the end of 2009, the park greenbelts area per capita in city has reach 9.71 square meters [28]. In addition, the annual new energy-saving building area is about 0.96 billion square meters, that is, there is 9 million tons of standard coal is saved and 23.4 million tons of CO2 emission is reduced. The national accumulative amount of energy-saving building area is 4.08 billion square meters and accounts for 21.7% of the total area.

In my opinion, the development prospect of low carbon city in China is perfect, and the cities will become a green and sustainable city in foreseeable future. Certainly, the supports of government and ordinary people are necessary, and the low carbon cities with low energy consumption of mining enterprises, the people of low-carbon lifestyle, low carbon traffic, low carbon buildings, and garden city characteristics will become the harmony family between human and nature in future.

### Forecasting for the non-fossil energy generation and energy consumption

At present, Chinese central government has established the Twelfth Five-year Plan, and the non-fossil energy sources are hoping to play the important role to improve the energy structure and to decline the unit energy consumption. Table 1 depicts the current and the predictive installed capacity in the future. In 2020, the non-fossil energy will account for more than 15% in the whole energy structure. Here, the installed capacities of the hydropower, nuclear power, wind power, and PV power are 350TW, 70 ~ 80TW, 150TW, and 50TW, respectively, and the total installed capacity will more than 550TW.

**Table 1:** **Non-fossil energy generation forecasting [Source:**[31-33]**]**

	**2011**	**2012**	**2015**	**2020**	
**Hydropower**	230TW	249TW	290TW	350TW	More than 550TW
**Nuclear power**	10.821TW	12.57TW	40TW	70 ~ 80TW	
**Wind power**	50TW	61TW	100TW	150TW	
**PV power**	3TW	5TW	21TW	50TW	
**Percent of the non - fossil energy (%)**	8	9.1	11.4	15	

With the rapid development of the economy and society, the growth of electricity consumption per capita and the total primary energy source consumption is inevitably to supply the ever-increasing demand of ordinary people, transmission, industry, etc. In order to realize the sustainable development goal, the saving energy and improved energy efficiency are necessary. According the predicted data, the GDP and the unit energy consumption will reach 20,000 billion dollars and 2.5 tons of standard coal per ten thousand dollars to compare with that of in 2005 is 2100 and 11.45. And the annual declining rate of the energy consumption is about 5.2% from 2005 to 2020. If don’t convert dollars to RMB, the GDP will reach 100,000 billion RMB and the ton of standard coal per ten thousand RMB is 0.5, and the annual declining rate of the energy consumption is about 4.1%. There are an obvious different in declining rate, and the fundamental reason is the exchange rate fluctuations. For example, the one dollar can exchange about 8.3 RMB in 2005, however, which only exchange about 6.1 RMB in 2013. The exchange rate is assumed to be 5 RMB to one dollar in 2020, as can be seen from Table 2. The predicted data is not equal to the actual data, the Chinese government has a lot of work to be done to realize the sustainable development and to conquer various barriers.

**Table 2:** **Energy consumption forecasting [Source:**[31-33]**]**

	**2011**	**2012**	**2015**	**2020**
**Electricity consumption per capita (KWH)**	3483	3600 ~ 3700	4000 ~ 4450	4800 ~ 5500
**The total primary energy source consumption (billion tons of standard coal)**	3.48	3.62	4	5
**GDP (billion dollars)**	7,300	8,400	11,000 ~ 12,000	20,000
**GDP (billion RMB)**	47,160	53,000	70,000	100,000
**Unit energy consumption (ton of standard coal per ten thousand dollars)**	4.7	4.3	3.63 ~ 3.33	2.5
**Unit energy consumption (ton of standard coal per ten thousand RMB)**	0.7379	0.683	0.5714	0.5
**Energy consumption declining rate (%) (converted into dollars)**	6	9	Annual average by 5.2 ~ 7.5 from 2012	Annual average by 8.3 ~ 10 from 2015
**Energy consumption declining rate (%) (no converted into dollars)**	9.6	7.5	Annual average by 5.45 from 2012	Annual average by 2.5 from 2015
**The exchange rate (one dollar to RMB)**	6.5	6.3	6	5

### Development barriers

Today, the development of low carbon city in China has come into the rapid stage, and the regard of common people and central government is increased. However, there are some policy barriers and financial barriers and habits barriers and technology barriers have obstructed the rapid development of low carbon city in China [34-39].

Firstly, the development policy of low carbon society has been established by the central government, but these policies are difficult to implement by local government due to the behalves and financial resources of local government are different. Thus there are different actual actions to realize the target of low carbon city. There are more than thirty provinces in China, and different provinces have different impetus because of different province hold different natural resource. For example, Shanghai has little fossil resources, but which have abundant financial resources and enough technologies and talents, that is, Shanghai government has big impetus to realize the low carbon city. On the contrary, some west and north provinces have enough fossil resources like Neimenggu and Shanxi province, but the financial resources and technologies and talents are deficient. Also, the financial resources mainly rely on the mining industry, which is high-energy consumption and high pollution, thus the impetus is little.

Secondly, the development of low carbon city needs billions of dollars. For example, the price of low carbon building is more than that of conventional building. Few people are willing to spend more money on housing because of current high housing prices in China. Besides, the study and introduction of low carbon technologies require large amounts of money, and the investment for small and medium-sized companies is so great that they are unable to withstand. As a conclusion, the financial support of government is necessary to improve the development of low carbon city in future.

Thirdly, Since 1978, the national reform and opening, most Chinese cities, have formed the habit of high-energy consumption, such as air conditioning, television and other household appliances typically use the standby mode, and the transportation mode of thousands of people is the airplane or private cars, and millions of people buy dozens sets of clothes per year, etc. So the low-carbon lifestyle is difficult to accept by the ordinary people in a short time. Certainly, the low-carbon lifestyle is the low carbon urban developmental important constituent, so it needs the government increased publicity and ordinary people to know enhancement.

Finally, the low carbon technology in China is not regarded by government and the experts and university and graduate school in the past years like clean coal technology, low carbon building material, renewable energy application, and so on. The technical investment is not enough which results in that it is impossible to exploit the key technology. Low carbon technologies for most of the domestic universities and research institute is unacquainted, certainly, the technology and talents reserves are scarce.

### Conclusion and recommendations

This paper presents the China’s energy structure, energy consumption per unit, Chinese urbanization, energy consumption status in urban, efforts and measures toward energy saving, and development prospect of low carbon city in China. In order to conquer more and more energy pressure in future and realize the sustainable development of nation, Chinese government and ordinary people have realized the important of low carbon development mode. With the technological advances and national attention, it can be predicted that the amount of low carbon city will rapid increase in foreseeable future. Certainly, there are some obvious policy barriers and economy barriers and technology barriers and habits barriers for the low carbon society at present, which need the strong financial and policy support of central and local governments and the changing of life style of ordinary people. The following measures are especially recommended in this regard:

1. The hortative policy of central government and local governments is necessary to realize the low carbon city in future.

2. Abundant fund should be invested in the research field of low carbon technology like clean coal technology, carbon emissions, building materials, and renewable energy application, etc. Certainly, the research of universities and graduate schools should be encouraged.

3. International cooperation between developed country and developing country should be encouraged to improve the domestic technology.

4. The central government and local governments should provide enough funds to help the development of low carbon building, city greening, and public transportation.

5. The tax reduction/exemption and policy initiatives are necessary to advocate the low carbon industry. At the same time, those high-energy consumption enterprises should be strictly punished to reduce the energy consumption per unit.

6. Low carbon life style should be advocated by the government.

## Competing interests

The authors declare that they have no competing interests.

## Authors’ contribution

All authors read and approved the final manuscript.
